# A Novel In Planta Enrichment Method Employing *Fusarium graminearum*-Infected Wheat Spikes to Select for Competitive Biocontrol Bacteria

**DOI:** 10.3390/toxins14030222

**Published:** 2022-03-17

**Authors:** Waldo Deroo, Larissa De Troyer, Fréderic Dumoulin, Sarah De Saeger, Marthe De Boevre, Steven Vandenabeele, Leen De Gelder, Kris Audenaert

**Affiliations:** 1 Department of Biotechnology, Faculty of Bioscience Engineering, Ghent University, 9000 Ghent, Belgium; leen.degelder@ugent.be; 2 Department of Plants and Crops, Faculty of Bioscience Engineering, Ghent University, 9000 Ghent, Belgium; larissa.detroyer@ugent.be (L.D.T.); kris.audenaert@ugent.be (K.A.); 3 Centre of Excellence in Mycotoxicology and Public Health, Department of Bioanalysis, Faculty of Pharmaceutical Sciences, Ghent University, 9000 Ghent, Belgium; frederic.dumoulin@ugent.be (F.D.); sarah.desaeger@ugent.be (S.D.S.); marthe.deboevre@ugent.be (M.D.B.); 4 Aphea.Bio, Technologiepark 21, 9052 Zwijnaarde, Belgium; steven.vandenabeele@aphea.bio

**Keywords:** *Triticum aestivum*, *Fusarium graminearum*, biocontrol, pathobiome, culture independent, microbiome engineering, successive passage, experimental enrichment, phyllosphere

## Abstract

This work introduces an alternative workflow for the discovery of novel bacterial biocontrol agents in wheat against *Fusarium* head blight (FHB). Unlike the mass testing of isolate collections, we started from a diverse inoculum by extracting microbiomes from ears of field-grown plants at grain filling stage. Four distinct microbial communities were generated which were exposed to 3 14-day culture-independent experimental enrichments on detached wheat spikes infected with *F. graminearum* PH1. We found that one bacterial community reduced infection symptoms after 3 cycles, which was chosen to subsequently isolate bacteria through limiting dilution. All 94 isolates were tested in an in vitro and in planta assay, and a selection of 14 isolates was further tested on detached ears. The results seem to indicate that our enrichment approach resulted in bacteria with different modes-of-action in regard to FHB control. *Erwinia persicina* isolate C3 showed a significant reduction in disease severity (*Fv*/*Fm*), and *Erwinia persicina* C3 and *Pseudomonas* sp. B3 showed a significant reduction in fungal biomass (cGFP). However, the mycotoxin analysis of both these treatments showed no reduction in DON levels. Nevertheless, *Pantoea ananatis* H3 and H11 and *Erwinia persicina* H2 were able to reduce DON concentrations by more than 50%, although these effects were not statistically significant. Lastly, *Erwinia persicina* H2 also showed a significantly greater glucosylation of DON to the less phytotoxic DON-3G. The bacterial genera isolated through the enrichment cycles have been reported to dominate microbial communities that develop in open habitats, showing strong indications that the isolated bacteria can reduce the infection pressure of *F. graminearum* on the spike phyllosphere.

## 1. Introduction

*Triticum aestivum* L., or bread wheat, is the world’s third most important food crop, with an estimated global gross production value of USD 187.7 billion. Exceeded only by rice and maize and followed by soybeans, wheat is a driver of global economies and contributes to about 20% of the total dietary calories and proteins worldwide [1,2].

Fusarium head blight (FHB) is a significant threat in wheat crops. Warm, wet summers with high relative humidity during flowering (anthesis) and early grain formation promote the infection and increase the risk of economic losses [3]. In addition to causing yield loss, FHB can result in the accumulation of mycotoxins in the infected grain, with detrimental effects for human and animal health.

*F. graminearum* is one of the main fungal species that causes FHB. Conventionally associated with southern European zones and warmer climates, this species has gradually adapted to more northern regions. This is due to global warming but also due to its ability to, unlike other FHB pathogens, reproduce sexually. *F. graminearum* is a homothallic species, meaning that in addition to asexual conidiospores, it regularly produces sexual ascospores, hence increasing its ability to generate more variability for adaptation and virulence [4]. As a result, *F. graminearum* plays a pivotal role in the FHB complex in wheat [5,6]. In grains, this fungal species is mainly responsible for the accumulation of the mycotoxin and virulence factor deoxynivalenol (DON).

The infection of wheat by *F. graminearum* is characterized by distinct stages. As spores land on the spikes they germinate on the anthers or inside the open florets. Extracellular hyphae advance between live host cells without causing visible disease symptoms, growing as an apoplastic biotroph. The symptomatic phase occurs when surrounding fungal hyphae enter the wheat cells via infection cushions. The parenchyma of the pericarp begin to break down, and *F. graminearum* readily grows both intra- and intercellularly throughout the entire kernel. The first visible symptoms occur as dark brown, water-soaked spots on the glumes of infected florets. This intracellular colonization of dead wheat cells depicts the necrotrophic phase of the fungus. The onset of the necrotrophic phase is also characterized by the activation of Tri genes, responsible for trichothecene and DON production [7,8]. Shortly after the colonization of the spikelet, the mycelium follows the vascular bundles of the node and rachis and propagates up through the wheat spike. The hyphae develop first within the conductive vessels and then grow into the surrounding tissue. In 1963, Schroeder and Christensen identified two types of genetic resistance to *Gibberella zeae* (i.e., *F. graminearum*). Type I resistance operates against initial infection and Type II against the the spread of the infection within the host [9]. The types of resistances were further expanded by Miller et al. (1985) and Mesterhazy et al. (1999), who identified resistance to toxin accumulation (Type III), resistance to kernel infection (Type IV) and tolerance (Type V) [10,11]. Tolerance could be detected at different resistance levels and demonstrated yearly differences in the field, even when the methodology was the same. This led to the nomination of resistance Type V, being independent of the other four types. These five types of resistances are well-established in literature and are widely accepted as the main types of genetic resistance against FHB in wheat genome [12,13,14,15].

The use of pesticides is a conventional agricultural practice. However, the efficacy of fungicide treatment, specifically triazoles, for FHB is variable and difficult to predict. Efficacy greatly depends on method and date of application of the product [16,17]. In addition, both scientific and public concerns are arising about the continued dependence on chemical fungicides, due to their contaminant environmental impact and the development of resistance in the pathogen population [18]. Consequently there is an urgency to develop non-chemical control strategies and more attention should be given to find alternative inputs of less toxic and less disruptive methods for the world’s staple food crops. Moreover, in the management of FHB the application of fungicides at sub-lethal concentrations can even trigger mycotoxin biosynthesis in *F. graminearum* [19].

The use of biocontrol agents (BCAs) to control FHB represents an alternative approach and may be used as part of an integrated pest management. The focus of this study is to isolate bacteria that can promote the type I resistance of the plant and reduce the infection pressure during the biotorphic phase of the fungus. Anthesis has been shown to be the most susceptible stage for initial FHB infection, resulting in high toxin levels [20]. By minimizing the infection pressure at this critical time point, biocontrol organisms could considerably reduce the mycotoxin accumulation of the harvested grains.

Biocontrol strategies have been extensively researched over the last decades; however, only a few BCAs are currently commercially available [21,22,23]. This is in part due to the complexity of legislation and the high cost of registration that prevent a large number of potential BCAs from reaching the market [23], but it is primarily a result of the variable performance of biocontrol strategies when assessed under field conditions [24,25]. This observation has been attributed to a combination of factors such as not reaching the threshold number of viable cells for the establishment of a plant–microbe interaction, an incompatible growth phase at the time of application and a rapid decline in the population after inoculation in an agro-ecosystem [26]. To counteract these factors, BCAs can be (i) formulated to supplement their natural shortcomings or (ii) selected for niche establishment, as well as their biocontrol effect, meaning that the interaction with the host plant must be robust so that they express a beneficial effect in simple systems as well as challenging environments. We hypothesise that this is because many studies focus solely on the direct antagonism between *F. graminearum* and the BCAs, resulting in strong effects in well-controlled environments but not in the variable conditions of agro ecosystems.

A prime example is the study by Chen et al. (2018), who screened more than 12,000 culturable bacterial isolates for antagonistic properties in a dual-culture assay, continuing in planta experiments of only the candidates that demonstrated a direct antagonism in vitro and, finally, obtained one potential BCA (*Pseudomonas piscium* ZJU60) with high antagonistic activity against FHB [27]. While this study might be broad in scope, the rate of successful isolates compared to the total screened was relatively low. This is also in line with the findings of Wang et al. (2015), who concluded that antagonistic inhibition in co-culturing assays showed low correlation with biocontrol efficacy [28].

Moreover many of the previous studies do not take into account the original habitat of the selected isolates. Meaning that, for example, soil-dwelling organisms, not suited for the nutrient-poor environment of the phyllosphere, are recruited as potential biocontrol agents against spike diseases. In a 2015 study, Wang et al. screened a total of 966 bacterial isolates for antagonism against *F. graminearum* in rhizosphere and phyllosphere conditions; however only 1 isolate (*P. fluorescens* LY1-8) was able to control *F. graminearum* in both habitats [28]. Notably, all the isolates that performed well in field trial against FHB were originally isolated from wheat ears.

The plant-associated microbiome consists of a few thousand bacteria, fungi and protists, comprising a community that colonizes the roots and above-ground plant parts. These microbes govern the specific niches of their host. In effect, plant physiology and performance strongly depends on the dynamics within their microbial community [29,30,31]. Under natural conditions, the two major groups of the plant microbiome are bacteria and fungi, and their interactions are paramount in shaping the environmental microbial communities and effect the fitness, colonization or pathogenesis of the interacting partners [27,32]. The observation that certain diseases might be caused, altered or suppressed by biotic factors other than the pathogen itself lead to the conjecture of the pathobiome [33].

Beneficial bacteria can degrade fungal virulence factors, induce plant systemic resistance or even produce volatile antifungal compounds [34,35,36]. A recent study has explored the role of the pathobiome in rice sheath rot, caused by the fungus *Sarocladium oryzae* [37]. The authors noted that virulence of *S. oryzae* was dependent on its ability to produce antibiotics as opposed to phytotoxins. Concluding that the fungus needs to produce antibiotics to defend itself against antagonistic rice endophytes to successfully colonize and infect the rice sheath, hereby observing a strong shift in the microbial community that correlated with disease virulence.

Our study aims to improve the rate of successful biocontrol isolates compared to the total screened. This is pursued by a culture independent experimental enrichment of the wheat spike through successive passages of the microbial community. The successive passage of an inoculum has proven to have a significant effect on the composition of the plant-associated microbiome in both culture-dependent [38,39] and culture-independent studies [40,41].

Experimental enrichment has also been demonstrated in a biostimulant study by De Zutter et al. (2021) [42]. When testing an in planta enrichment concept, enabling simultaneous microbial selection for P solubilization and rhizosphere competence, they found a shift in bacterial populations that co-occurred with an improved P status of inoculated maize grown under P-limitation. After the third consecutive enrichment in the rhizosphere community comprised bacterial groups that are typically associated with plant growth promotion (specifically P-solubilization). They concluded that this P-solubilizing consortium could be used as a valuable source for the in vitro isolation of single strains, to be subsequently evaluated in plants.

In our research, four lines of uncultured phyllosphere extracts are generated from field-grown plants. These four bacterial communities are successively passed over a set of cut-spikes under biotic pressure of *F. graminearum*, resulting in three consecutive enrichments. To see if the different bacterial communities exercise an anti-fungal effect, disease symptoms are monitored throughout the cycles, hereby testing the hypothesis that phyllosphere microbiomes can adapt to the plant host environment and reduce the disease pressure of *F. graminearum*. The microbial population that colonizes the spike phyllosphere and best reduces FHB symptoms will be subsequently tested in assays with increasing ecological relevance, and the success rate of the isolates will be monitored for every assay.

## 2. Results

### 2.1. Optimisation of Inoculant Carrier for Maximum Adherence to the Spike

**Figure 1:**
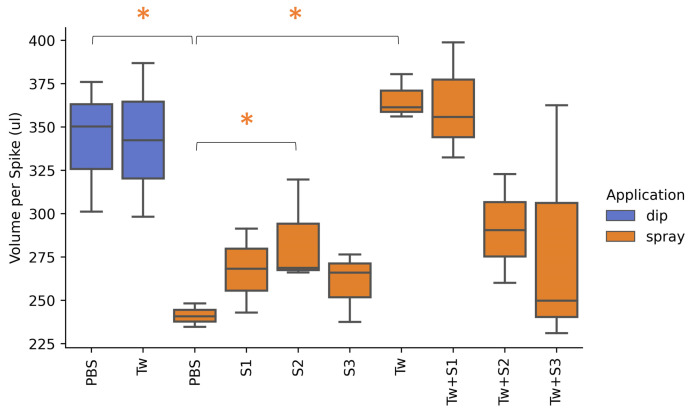
Optimisation of Carrier for Maximum Adherence to the Spike. The volume adhering to the spike after spray application was compared for eight different carrier mixes (orange). Adherence of PBS and Tw was tested via two different application methods: dipping (blue) and spraying (orange). Treatments that showed a significant difference are marked with an asterisk (*p* < 0.05).

The adherence of eight different carrier mixes (Table 1) was assessed using two different application methods. When comparing dipping applications, the PBS and PBS + Tween treatments resulted in similar adherence for the spike. The calculated amount of carrier mix adhesion per spike varied between 298 µL and 387 µL, with an average around 340 µL for both treatments (Figure 1). When the PBS treatment was sprayed, however, the adherence to the spike was significantly reduced. Here, the average amount of carrier mix decreased to 241 µL per spike. The spray application of PBS + 0.05% Tween 20 resulted in a significantly higher adherence compared to spray application of PBS without surfactant. Addition of tween resulted in an average of 366 µL adhering to the spike when sprayed. This was the highest volume of all the treatments throughout the experiment. There was no significant difference between a dip and spray application of PBS + 0.05% Tween 20.

Subsequently, three commonly used stickers were tested to see if they could further promote the adherence. When the stickers were added to the carrier mix without inclusion of tween, S2 (MC) significantly increased the adherence compared to the spray application of PBS. This additional volume, however, was small, with an average increase of 44 µL per spike (+18%). Stickers S1 (CMC) and S3 (Metasperse^TM^) also increased the adherence by 26 µL (+11%) and 19 µL (+8%), respectively; however, this was not significant. The addition of a sticker to the carrier mixes that contained Tween did not result in a higher adherence to the spikes. Moreover, carrier mix Tw + S2 and Tw + S3 resulted in a lower adherence with a decrease of 75 µL (−20%) and 85 µL (−23%), respectively. As a result, all future experiments were conducted using a carrier consisting of PBS + 0.05% Tween 20 (designated Tw in Table 1). This carrier was utilized for the bacterial application as well as the inoculation of *F. graminearum* via spray application in the enrichment cycles and the detached spike assays.

### 2.2. In Planta Enrichment for Biocontrol Isolates Persisting on the Spike

**Figure 2:**
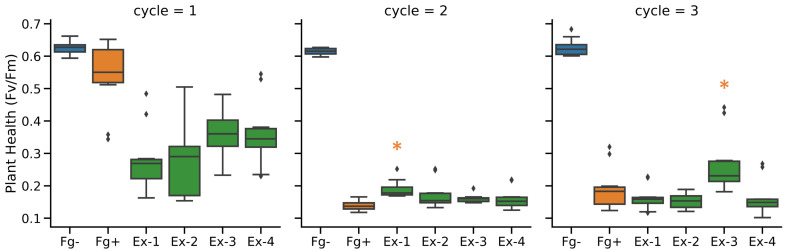
In Planta Enrichment Cycles. The effect of the 4 microbial communities on the disease development of *F. graminearum* in a detached spike assay, through experimental enrichment in 3 successive passages. Disease severity was assessed by measuring photosystem II efficiency (*Fv*/*Fm*). Treatments that showed a significant difference compared to the infected control are marked with an asterisk (*p* < 0.05).

To assess the disease progression of *F. graminearum* (Fg) during the enrichment cycles, multi-spectral images were taken at 14 days post inoculation (dpi). The *Fv*/*Fm* ratio was selected to quantify the infection of *F. graminearum*. The results are shown in Figure 2. For the first cycle, four microbial communities (Ex-1, Ex-2, Ex-3 and Ex-4) were generated from field-grown wheat and spelt varieties and were each applied onto a cut-spike assay. The disease pressure for all treatments in this cycle was relatively low; however, at 14 dpi, there was a significant difference between the uninfected control (Fg−) and treatments Ex-1, Ex-2, Ex-3 and Ex-4. Dunn’s test of multiple comparisons using rank sums and *p*-values adjusted with the Benjamini–Hochberg method resulted in a significantly lower *Fv*/*Fm* for all of the extract treatments compared to the uninfected control (*p* < 0.05). The infected control (Fg+) showed a reduction in the *Fv*/*Fm*; however, this did not significantly differ from the uninfected control (Fg−). Due to the large variability in the disease symptoms in this cycle, no significant difference was observed between the infected control and the extract treatments. In cycle 2, an increased disease pressure was observed. Here, all treatments, including the infected control, were significantly different from the uninfected control (*p* < 0.001). When comparing the extract treatments to the infected control, all the extracts resulted in a higher *Fv*/*Fm*. However small, Ex-1 resulted in a significant improvement in the *Fv*/*Fm* (*p* = 0.03). In the final cycle, cycle 3, Ex-3 showed a clear positive effect on the *Fv*/*Fm* after 14 days. This improvement was significant when tested one-sided (*p* = 0.026) compared to the infected control. Moreover, when tested two-sided, Ex-3 resulted in a significantly better *Fv*/*Fm* signal than treatments Ex-1, Ex-2 and Ex-4, with a *p*-value < 0.015 for all comparisons.

### 2.3. Reduction in Mycelial Growth in a Dual Culture Assay

**Figure 3:**
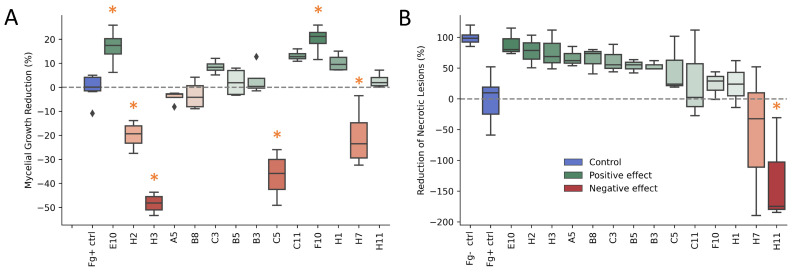
Assessment of the biocontrol potential of bacterial isolates. (**A**) Assessment of the biocontrol potential of bacterial isolates. (**A**): Mycelial growth reduction in an In vitro dual culture assay. (**B**): Reduction of necrotic lesions in a detached leaf assay. Treatments that showed a significant difference compared to the infected control are marked with an asterisk (*p* < 0.05).

Subsequent to the in planta enrichment cycles, Ex-3 was selected for isolating bacteria present on the spike. With a similar methodology to the microbiome extract, the spikelets of Ex-3 were selected, and the spike microbiome extract was used as the starting suspension for limiting dilution, as described in Section 4.7. In total, 94 isolates were selected and preserved in a glycerol stock at −80 °C. Next, a dual culture assay was carried out for all 94 isolates to test for growth-inhibiting activity of *F. graminearum*. The mycelial growth reduction in all treatments is presented in Figure 3A. None of the isolates resulted in visible inhibition zones of the fungus. However, 37 of the tested isolates registered smaller mycelial growth diameters compared to the control treatment (Fg+ ctrl). Furthermore, when comparing all 95 treatments to the infected control, four of the isolates showed a significant reduction of mycelial growth in vitro. These isolates were F10, E10, B1 and F11 with reductions of 20%, 17%, 16% and 16%, respectively. Pairwise comparisons using Dunnett’s test for multiple comparisons with the control (alternative hypothesis: two sided) resulted in the respective *p*-values of < 0.001, 0.006, 0.012 and 0.015. In contrast, 14 of the isolates significantly promoted the mycelial growth compared to the control. These were H3, G7, C5, D2, G12, C4, E8, G2, H7, C12, H2, G5, E7 and C7. Pairwise comparisons using the same Dunnett’s test resulted in *p*-values <0.001, for H3, G7, C5, D2, G12, C4, E8, G2, H7, C12 and H2 and 0.002, 0.008 and 0.017 for G5, E7 and C7, respectively.

### 2.4. Reduction in Necrotic Lesions in a Detached Leaf Assay

An in planta experiment of the 94 isolates was conducted through a detached leaf assay. Disease assessment by multi-spectral imaging took place 3 days after inoculation. Comparing the *Fv*/*Fm* image of all 94 isolates to the infected control (Fg+ ctrl), 59 of the isolates resulted in a higher average *Fv*/*Fm* value, in effect reducing the development of necrotic lesions on the leaf (Figure 3B). In spite of their positive effect on the leaf, none of the treatments produced a significant effect over the infected control. Based on pairwise comparisons using Dunnett’s test for the comparison of all 95 treatments with the infected control (alternative hypothesis: two sided), only the uninfected control (Fg− ctrl) produced a significantly higher *Fv*/*Fm* ratio. In contrast, isolate H11 resulted in a significant lower *Fv*/*Fm* ratio and increased the disease symptoms by 130% (*p* = 0.010). Despite the disease reduction not being statistically significant, 9 isolates were able to diminish the development of necrotic lesions by >50%. These isolates were E10, H2, H3, A5, B8, C3, B5 and B3, with respective reductions of 90%, 78%, 76%, 67%, 65%, 63%, 57%, 54% and 53% in the detached leaf assay. Comparing these results to the in vitro reduction in mycelial growth, little correlation is observed. The scatter is visualised in Supplementary Figure S1. The measured parameters from both assays show approximately equal covariance, and the data are centered. A positive effect in the in vitro assay of an isolate was not predictive for the positive effect observed in planta. This result is consistent with the findings of Wang et al. (2015), who concluded that antagonistic inhibition in co-culturing assays showed low correlation with biocontrol efficacy [28].

### 2.5. Identification of Bacterial Isolates

Before advancing to an in planta detached spike assay, a selection of the most promising isolates was made based on their efficacy in vitro and their efficacy in the detached leaf assay. All isolates that resulted in a reduction in lesion development greater than 50% in the detached leaf assay were selected for identification and further testing of their biocontrol properties. In addition, isolates that resulted in a reduction in mycelium growth greater than 10% in vitro and that showed a positive effect in the detached leaf assay were also selected. Accordingly, isolates A5, B3, B5, B8, C11, C3, C5, E10, F10, H1, H2 and H3 were cultured, identified and tested in a detached spike assay. A visual overview of the selected isolates can be found in Supplementary Figure S1. Furthermore, two isolates were selected as controls. These were H7, since it promoted the development of *F. graminearum* in vitro, as well as in the detached leaf assay, and H11, since it showed a strong promotion of necrotic lesions in planta. All 14 isolates were identified through Sanger sequencing, and the 16S rRNA gene region was amplified using universal primers as described in Section 4.8. The identities of the 14 isolates are presented in Table 2. We found that 11 of the 14 identified isolates belonged to the Erwiniaceae family, and only 2 belonged to the Pseudomonadaceae, while 1 was inconclusive. *Pantoea ananatis* was the most abundant species, comprising half of the identified isolates. *Erwinia persicina* was isolated three times, two undefined *Pseudomonas* spp. and one *Erwinia aphidicola* were also isolated. A full discussion of the isolate identities and their biocontrol effects are described below in Section 2.8.

### 2.6. Reduction in Disease Severity in a Detached Spike Assay

**Figure 4:**
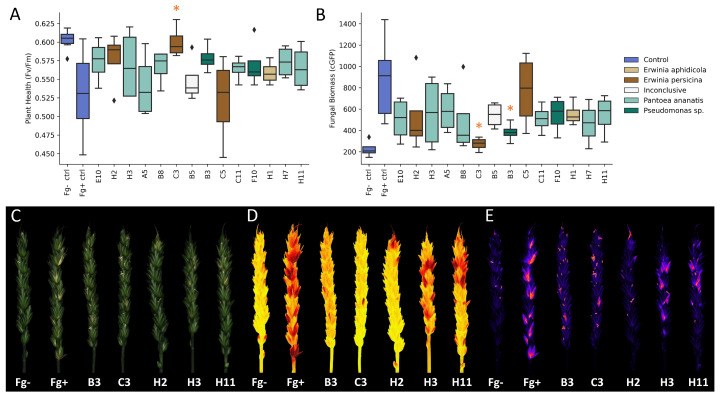
Reduction of disease severity in a detached spike assay (**A**): Disease severity was assessed by measuring photosystem II efficiency (*Fv*/*Fm*). (**B**): Quantification of mycelial growth on the spike was assessed by measuring the GFP signal, which was corrected for autofluorescence of the spike (cGFP). Treatments that showed a significant difference compared to the infected control are marked with an asterisk (*p* < 0.05). Multispectral imaging of selected treatments, (**C**): color image, (**D**): *Fv*/*Fm*, (**E**): cGFP.

To asses the biocontrol potential of the selected isolates in a ecologically relevant habitat, the bacteria and fungus were inoculated onto a detached wheat spike via spray application. Through multi-spectral imaging, disease severity was measured at 7 dpi using the *Fv*/*Fm* ratio (Figure 4A,D). Apart from isolate A5 and C5, all bacterial treatments were able to reduce the disease pressure on the spike. Moreover, among the bacterial treatments, isolate C3 also showed a significant increase in *Fv*/*Fm*, effectively reducing the occurrence and severity of the infection. A GFP-tagged *F. graminearum* strain was used to quantify mycelial growth on the spike by measuring the GFP signal, which was corrected for the autofluorescence of the spike (cGFP) (Figure 4B,E). Parallel to the positive effect on the *Fv*/*Fm*, a decrease in the cGFP signal was observed for all isolates except C5. Pairwise comparisons using Dunnett’s test for multiple comparisons with the control revealed that isolates C3 and B3 were able to reduce the cGFP signal, and thus mycelial growth on the spike, significantly by 92% (*p* = 0.001) and 74% (*p* = 0.013), respectively. Isolates H7, B8 and E1 also showed a strong effect on the mycelial growth, with reductions of 62%, 58% and 56%, respectively; however, these results were only significant when considered one-sided (*p* = 0.003, *p* = 0.038, *p* = 0.046).

### 2.7. Reduction in Mycotoxin Production in a Detached Spike Assay

**Figure 5:**
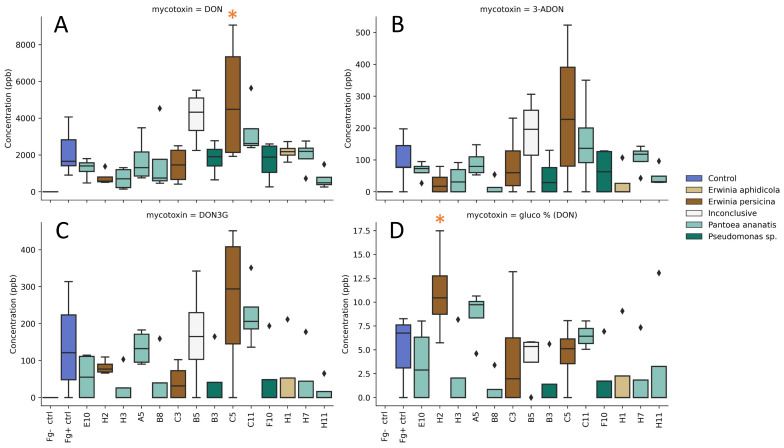
Effect of the bacterial isolates on the mycotoxin production in a detached spike assay, (**A**): DON, (**B**): 3-ADON, (**C**): DON-3G and (**D**): ratio of glucosylated DON. All concentrations are shown in µg/kg (ppb). Treatments that showed a significant difference compared to the infected control are marked with an asterisk (*p* < 0.05).

The effect of the isolates on the mycotoxin production of *F. graminearum* was assessed by analysis of the infected spikes from the detached spike assay at 7 dpi. The presence of the type B trichothecenes DON, 3-ADON and 15-ADON was measured. In addition, DON-3G, which is a detoxification product of DON synthesized by the plant, was measured, and finally, ZEN and derivatives α-ZEL and β-ZEL were measured through LC-MS/MS. A full rendering of the mycotoxin contents can be found in Supplementary Figure S2. DON levels for the infected control (Fg+ ctrl) had a mean value of 2160 µg/kg. In the uninfected control (Fg− ctrl), DON was not present. Among the bacterial treatments, three isolates resulted in an increased average DON concentration over the infected control (Figure 5A). These were C5, B5 and C11 with respective concentrations of 5000, 4100 and 3320 µg/kg. Of these treatments, C5 resulted in a significant increase (*p* = 0.017). Isolates A5, B3, B8, C3, E10, F10 and H1 showed a small decrease in the mean DON concentration compared to the infected control. Lastly, H11, H2 and H3 reduced the DON concentration by more than 50%, with respective mean concentrations of 680, 770 and 720 µg/kg. Despite these large reductions, they were not significantly different from the infected control due to the large interspike variability of the control. Similar observations were made in regard to 3-ADON, although there were no significant differences (Figure 5B). ZEN and 15-ADON were present in few samples and were only present in very low concentrations in the infected control (Supplementary Figure S2). To reduce the phytotoxicity of DON, plants have developed detoxification strategies. The glucosylation of DON is one of the best-described strategies, converting DON to the less phytotoxic DON-3G, after which it is directed to the vacuole [43]. The presence of DON-3G within the treatments showed a strong correlation with the DON concentration, although no significant differences in DON-3G content were observed (Figure 5C). To examine whether the presence of the isolates affected the plants capacity to glucosylate DON, the ratio of DON-3G over total DON was calculated. In this experiment, the infected control showed a glucosylation ratio of 5%. This is consistent with the results of Tan et al. (2021), who observed similar rates in infection experiments of wheat ears with *F. graminearum* PH-1 [44]. The examination of bacterial treatments where DON-3G was present revealed a high glucosylation ratio for treatment H2. When spikes were inoculated with this isolate prior to infection of *F. graminearum*, 11% of the DON was present in the glucosylated form (Figure 5D). The increase mainly results from the fact that less DON had been formed in the H2 treatment. Notwithstanding, the ratio of glucosylated DON was significantly greater compared to the infected control (*p* = 0.048).

### 2.8. Overall Comparison of Isolate Performance

To compare the success rates of the isolates in the three assays a Fisher’s least significant difference analysis (LSD) was performed for each. Isolates that showed a significant positive effect compared to the infected control were counted. In the in vitro co-inoculation assay, 10 isolates showed a significant reduction in mycelium growth. Suggesting 10 out of the 94 tested isolates possessed potential biocontrol properties in vitro, resulting in a success rate of 11%. Among these, two *Pantoea ananatis* isolates (E10, C11) and one *Pseudomonas* sp. isolate (F10) were identified. For the detached spike assay we found that nine isolates showed a significant increased *Fv*/*Fm* ratio compared to the infected control. Consequently, 9 out of the 94 tested isolates showed potential biocontrol properties in the detached leaf assay, resulting in a success rate of 10%. Among these, four *Pantoea ananatis* isolates (E10, H3, A5, B8), two *Erwinia persicina* isolates (H2, C3) and one *Pseudomonas* sp. isolate (B3) were identified. LSD analysis of the detached spike assay resulted in a significant improvement in the *Fv*/*Fm* for three isolates; these were isolate *Pseudomonas* sp. B3, *Erwinia persicina* C3 and *Erwinia persicina* H2. *Pseudomonas* sp. B3 and *Erwinia persicina* C3 also showed a significant reduction in mycelial growth (cGFP). Isolate *Erwinia persicina* H2 did not confirm in the cGFP range because one of the spikes showed a substantially higher cGFP signal than the others in the treatment. This greatly influenced the treatment’s average, resulting in high inter-spike variability and a non-significant difference compared the infected control. For the purpose of this comparison, we removed this outlying data point. This allowed us to identify 3 isolates that showed potential biocontrol properties out of the 14 tested, improving both plant health (*Fv*/*Fm*) and reducing the fungal presence (cGFP) in a detached spike assay, resulting in a success rate of 21%. Lastly, when comparing the DON concentrations of the bacterial treatments to the infected control using LSD analysis, isolates *Pantoea ananatis* H11, *Erwinia persicina* H2 and *Pantoea ananatis* H3 significantly reduced the presence of DON, suggesting that 3 out of the 14 tested isolates (21%) possessed potential to control the mycotoxin production in the spike when infected with *F. graminearum*. Notably, only isolate *Erwinia persicina* H2 was able to reduce mycotoxin levels while also reducing the visual infection symptoms (*Fv*/*Fm*). In contrast, isolate *Pantoea ananatis* H3 did not reduce visual infection symptoms; however, it did result in a lower mycotoxin content. Finally, isolate *Pantoea ananatis* H11 showed a strong promotion of necrotic lesions in the detached leaf assay and was included in the detached spike assay as a control. However, no increased disease symptoms were observed in the detached spike assay. Furthermore, this isolate was able to reduce DON concentrations in the treated spikes.

A full correlation analysis comparing the different parameters, measured in the dual-culture, detached leaf and detached spike assays can be found in Supplementary Figure S3. When comparing the performance of the individual isolates over all the assays, it becomes apparent that none of the isolates that performed well in the in vitro assay showed a reduction in disease symptoms in the detached spike assay. In contrast, isolates *Pseudomonas* sp. B3, *Erwinia persicina* C3 and *Erwinia persicina* H2, which performed well in the detached leaf assay, resulted in a reduction in disease symptoms in the detached spike assay. Moreover, isolate *Erwinia persicina* H2 also showed a reduction in DON concentration.

The ratio of potentially interesting isolates for the in vitro and detached leaf assay was approximately the same. In these assays, around 10% of the tested bacteria showed a potential biocontrol effect. When moving to the more complex and ecologically relevant system of the detached spike assay, we found that around 20% of the bacteria showed a potential biocontrol effect, improving both plant health (*Fv*/*Fm*) and reducing the fungal presence (cGFP). Additionally, we found that 20% of the tested bacteria were able to reduce the DON concentration in a detached spike assay. Notably, only one isolate (*Erwinia persicina* H2) was able to improve the plant health (*Fv*/*Fm*) and reduce the mycotoxin content.

## 3. Discussion

Fusarium head blight (FHB) in wheat production fields can experience annual fluctuations due to variations in weather. However, when infection pressure is high and climate conditions are conducive, the consequences to yield and quality are disastrous. Total yield decreases and more importantly, mycotoxins accumulate in the grains, making them unsuitable for consumption. Conventional agricultural practice is to treat the disease with fungicide, but there is a need for green alternatives, as both scientific and public concerns arise over the continued reliance on chemical crop protection due to their polluting environmental impact and the development of resistance in the pathogen population [18]. Biocontrol strategies have been extensively researched over the last decades since they could provide a long-term, cost-effective and safe solution. Numerous studies have produced a non-exhaustive list of biocontrol agents that have been shown to reduce FHB symptoms by *F. graminearum* (For a review, see [22,23]). However, only a small number of the biocontrol agents are currently available as a commercial product for field use. We hypothesise that this is because many studies focus solely on the direct antagonism between *F. graminearum* and the biocontrol agents (BCAs) [45,46], resulting in strong effects in well-controlled environments but do not demonstrate the same effect in the variable conditions of agro-ecosystems. This likely is in part due to their inability to colonize and survive on the spike phyllosphere, where nutrients are much scarcer and microbes are exposed to harsh environmental stresses such as drought and UV-radiation [47,48].

In our study, we tested a culture-independent method to enrich a starting microbial community, generated from ears of field-grown wheat, for bacterial strains that can proliferate on the spikes and survive over 3 14-day passages. This is a novel approach, in contrast to the numerous studies that start from the large-scale assessment of microbial collections for in vitro antagonism by co-culturing, resulting in very few isolates that show biocontrol activity in field conditions out of the thousands that were tested [27,28]. Moreover, many of previous studies do not take into account the original habitat of the selected isolates. Meaning that, for example, soil-dwelling organisms, not suited for the nutrient poor environment of the phyllosphere, are recruited as potential biocontrol agents against spike diseases. In addition, little to no attention is given to the potential of the bacterium to survive common agricultural actions such as formulation and application methods. The methodology used in our study facilitates later processing and formulation as they expose the bacteria to the shear forces of a spray application and to commonly used concentrations of surfactant.

Through successive passages of the microbial community of *T. aestivum* var. AB-11 on detached wheat spikes under biotic pressure of *F. graminearum*, we found a significant reduction in disease severity after there cycles, whereafter 94 bacterial isolates were obtained by limiting dilution. Through least significant difference analysis, 11% of the isolates showed a significant reduction in mycelium growth in vitro, while 10% significantly increased plant health in a detached leaf assay. A selection of 14 of the 94 isolates was tested in a detached spike assay, in identical conditions as the enrichment cycles, where 21% of the isolates showed a significant reduction in disease severity and an equal percentage significantly reduced the mycotoxin deoxynivalenol (DON) in the spike. Notably, the different isolated bacteria showed relatively low genetic diversity on a family level; however, they did result in various modes-of-actions that reduced the disease pressure of *F. graminearum* in planta. This observation suggests that the enrichment cycles of the microbial community enrich for antagonism and not for a specific mode-of-action.

Two isolates that reduced the DON mycotoxin concentration in a detached spike assay (H11 and H3) were identified as *Pantoea ananatis*. This species has previously been described as an unconventional plant pathogen, occurring in diverse ecological niches but also contributing to growth promotion in potato and pepper [49]. It has also been described as a common endophytic bacterium of rice seeds, seedlings and adult plants and was even described as a hyperparasite of wheat leaf rust (*Puccinia graminis* f. sp. *tritici*) [50,51,52]. Yoshida et al. (2006) revealed that when this bacterium inhabited wheat heads, it produces quorum-sensing-related signal molecules, regulating exopolysaccharide biosynthesis and biofilm formation [53].

Isolates C3 and H2 both reduced the disease severity and presence of *F. graminearum* in the detached spike assay. Moreover H2 was able to significantly reduce the DON mycotoxin concentration in the spike. Both isolates were identified as *Erwinia persicina*. The genus *Erwinia* is considered to be a plant-phytogenic Gram-negative bacterium that causes fire blight and the wilting of leaves [54]. However, members of the genus have been identified as biocontrol agents of *Fusarium culmorum* and *Puccinia graminis* f. sp. *tritici*, as well as choline-metabolizing microbial strains, able to significantly reduce the disease severity of *F. graminearum* on wheat under field conditions [55,56]. The species *E. persicina* has also been reported to produce large quantities of acidic exopolysaccharide, involved in early stages of biofilm development [57]. The rapid composition of such a biofilm on the wheat spike during anthesis could possibly construct a barrier that is adverse for the spore germination and reduce initial infection pressure of *F. graminearum*. In 2014, Zhang et al. described multiple new legume hosts of *E. persicina* and its ability to be transmitted to the seed, possibly aiding their natural continuation [58]. A recent study identified barley (*Hordeum vulgare*) as a new host of *E. persicina*, causing pink coloration of the seeds that ultimately led to quality loss [59]. There is a lot to gain from rapid colonization of the wheat spike by *E. persicina* when infection of *F. graminearum* is imminent. However, the interaction between an endophyte and its host is subject to various environmental conditions, and there are many parameters that can cause the interaction to shift along the mutualism parasitism continuum [60]. Althernatively, as Hallmann (1997) concluded, spatially limited colonization is characteristic of endophytic bacteria and probably a major factor in differentiating endophytic bacteria from plant pathogens [61]. This is reiterated by the findings in our study that seem to suggest that biocontrol effects are strain-specific. One example is the consistent beneficial effect of isolates C3 and H2, while isolate C5 was the worst-performing isolate in the detached spike assay; however, these all belong to the same species complex of *E. persicina*.

Isolate B3 was able to significantly increase the plant health (*Fv*/*Fm*) and reduce mycelial growth (cGFP) in the detached spike assay. This isolate was identified as a *Pseudomonas* sp., while the *Pseudomonas* genus is one of the most abundantly reported genera in biocontrol studies against FHB [27,28,46,48,56,62].

Members of the genera *Pantoea*, *Erwinia* and *Pseudomonas* have been reported to thrive in and dominate microbial communities that develop in open habitats, such as the phyllosphere [63]. Furthermore, these genera have been described as “microbial weeds”, who out-grow competitors via traits such as robust tolerances to habitat-relevant stress parameters, highly efficient energy-generation systems and exceptional abilities to sequester and store resources [63]. These strategies are ecologically significant, as they occupy the same niche as phyllosphere pathogens [64] and support the hypothesis that fast-growing, ear-colonizing bacteria could out-compete *F. graminearum* spores for nutrients and space when they land on the spikes. As a result, these microbial communities could reduce the initial infection pressure, much like a wheat genetic type I resistance, resulting in a lower infection rate and lower accumulation of mycotoxins in the field. One might even suggest that wheat genotypes that possess a strong type I resistance against *F. graminearum* are innately capable to attract these fast-colonizing microbial communities at time of anthesis, hereby effectively showing a level of resistance against the fungal pathogen. For isolates that reduced the mycotoxin content in the spike, we observed a strong parallel with the type III resistance, which enables the plant to manifest resistance to toxin accumulation. Thirdly, resistance type V, tolerance, which is reported to demonstrate yearly differences within the same wheat cultivar, could be a result of changing microbial communities residing on the spike at time of infection and disease development. However, future studies will have to examine the circumstances under which these genotypic and microbial type I, type III or type V resistances can overlap.

In this study, the effect of the bacteria on the biosynthesis of mycotoxins was not directly investigated. However, through cGFP measurements, the DON concentration could be linked to mycelial growth on the spike. This suggests that for isolate *E. persicina* H2, the reduction in DON in planta can be attributed to the reduction in the fungal development in planta. On the other hand, we found two isolates, *Pseudomonas* sp. B3 and *Erwinia persicina* C3, that significantly reduced the mycelial growth on the spike; however, they did not reduce the DON concentration. This might indicate that although the disease development is slowed down, the bacteria are not capable of completely inhibiting the fungus, possibly creating a stress factor for the pathogen and stimulating mycotoxin biosynthesis. The impact of a fungal stress factor has previously been linked to an increased DON production by *F. graminearum* [5,19,35]. We also observed two isolates (*Pantoea ananatis* H11 and H3) that did not reduce the mycelial growth on the spike, but show a strong reduction in the DON concentration. It is unclear how these isolates interact with the fungus, but it has recently been reported that bacteria could be able to influence the mycotoxin biosynthesis by inhibiting *Tri*-gene expression [65]. Moreover, some bacterial taxa are known to metabolize DON [66]. Lastly, interaction with the plant defense mechanism also has the potential to reduce disease pressure. It has been reported that wheat can detoxify DON to the less phytotoxic DON-3G and that biocontrol agents can increase the plant’s capacity to glucosylate DON [44,67]. The presence of a high concentration of DON-3G in the infected spikes treated with *E. persicina* H2 could point to a positive feedback on the plant defense mechanism.

An increased interest in the pathobiome has emerged in recent years observing the intricate host–pathogen associations, as well as microbe–microbe associations in phytobiomes and agroecosystems. Pathogens not only deal with immune responses during host colonization but also encounter other microbes [68]. A change in the microbial community that is present in the phyllosphere at different levels of virulence has recently been observed by Peeters et al. (2021) [37]. It is also reported that pathogen stress can have a greater impact on the microbial community than the plant genotype, shaping specific microbial communities and interactions that would not be present on healthy plants [69].

Our findings support the idea that culture-independent experimental enrichment of the microbial population of the wheat spike under an infection pressure can select for bacteria with potential biocontrol properties. The identification through 16S rRNA gene sequencing shows strong indications that the isolated bacteria have an advantage in the competition for niche and nutrients on the spike phyllosphere. Furthermore we observed antagonism through different modes-of-action that overlap with well established types of genetic resistance in wheat genotypes, suggesting a microbial community enriched for bacteria that show antagonism against *F. graminearum* but not for a specific mode-of-action. Finally, these different modes-of-action could pave the way to investigate the combination of different isolates, creating synthetic consortia and their combined effect against *F. graminearum*.

## 4. Material and Methods

### 4.1. Optimisation of Inoculant Carrier for Maximum Adherence to the Spike

For the detached spike experiment, 8 different carrier mixes for microbial inoculation were tested and assessed for adherence to wheat spikes (Table 1). A phosphate buffer saline solution (PBS) of 15 mM was used as solvent. All carrier mixes were applied via spray application. Carrier mixes PBS and Tw were also applied via dip application. Spray application was performed using a spray paint brush (Fengda BD-138 Airbrush pistol) and an over-pressure of 1 to 1.5 bar. For every treatment, the respective mix was sprayed over 9 spikes of equal size until runoff. Spikes were sprayed from 5 cm distance (nozzle to spike) on both sides and left to dry for 5 min. For the dip application, spikes were fully submerged in the carrier mix for 5 s and left to dry for 5 min. Thereafter, the 9 spikes were washed per 3, resulting in 3 replicates per treatment. Spikes were washed in 50 mL reverse osmosis (RO) water and shaken vigorously by hand for 30 s. Finally, 100 µL was loaded into a 96-well plate, and samples were measured in a micro-plate reader at 550 nm. A standard dilution of RO water and water soluble tracking dye was made and used to calculate the total amount a liquid adhering to the spikes.

### 4.2. Spike Microbiome Extraction from Field

Spikes were collected from different wheat and spelt cultivars from a grain variety trial in Evergem, Belgium (by courtesy of AlterGrain project, HOGENT). Within the trial, a visual selection was made of healthy, green spikes within a zone of higher disease pressure. Sampling took place at grain-filling stage (GS83). Based on these criteria, three wheat cultivars and one spelt cultivar were selected for sampling; these were *Triticum aestivum* L. var. Mentor (Ex1), *T. spelta* var. Sérénité (Ex2), *T. aestivum* var. AB-11 (Ex3) and *T. aestivum* var. Ponticus(Ex4). Samples were kept at 4 °C until further processing.

A spike microbiome extraction was performed the same day on the fresh plant material. Per sampled cultivar, 5 fully developed spikelets from 4 different spikes were randomly selected, for a total of 20 spikelets. The 20 selected spikelets were crushed for 2 min in a sterile, pre-cooled mortar with 20 mL PBS (4 °C). Crushed material was transferred to a sterile falcon and shaken vigorously by hand for 30 s, after which, a plastic syringe was used to extract all the liquid from the crushed spikelets. The liquid was passed over a 40 µm cell strainer. Finally, the suspension was centrifuged for 20 min at 5220 g (4 °C), supernatant was discarded, pellet resuspended in 1 mL PBS and kept on ice until application. Just before application, 15 mL of carrier mix was added to the extract. All applications of bacteria and inoculations of *F. graminearum* conidiospores were carried out using PBS + tween 20 (0.05%) as carrier mix (designated Tw in Table 1).

### 4.3. Fungal Growth, Production and Isolation of Conidiospores

*Fusarium graminearum* PH-1 [70] was used for the infection assays. This strain was previously GFP-transformed by Tan et al. [5]. The transformant of *F. graminearum* PH-1 had no difference in fitness and/or virulence compared to the wild-type strain. *F. graminearum* PH-1 was grown on PDA for 14 days at 21 °C under a regime of 12 h of dark and 12 h of combined UVA and UVC light (2 × TUV 8 W T5 and 1 × TL 8 W BLB; Philips, Amsterdam, The Netherlands). Conidiospores were harvested by adding a solution of sterile 0.05% Tween 20 to the PDA plates and rubbing the mycelium with a Drigalski spatula. A 40 micron cell strainer was used to filter the conidia and remove the mycelium. Spores were counted using a Bürker counting chamber and suspension was diluted to a final concentration of $5\times 10^{5}$ spores/mL. This suspension was used for inoculations on both wheat leaves and ears.

### 4.4. Detached Spike Assay

Summer wheat (*T. aestivum* var. TYBALT) was grown in pots in the greenhouse until ear development. Plants were grown from seed in a commercial potting soil, and mineral fertilizer was added to ensure optimal development. Ears were collected at time of flowering (GS61) or shortly after (1–2 days). The time of flowering was determined when anthers were visible and flowering was complete to the top of the ear. On the day of the experiment, ears were cut from the plant and placed into commercial porous foam (OASIS${}^{®}$ Floral Foam Maxlife). The ears were kept in a nursery box (IKEA VÄXER) and placed in a controlled environment (day/night: 16 h/8 h, 24 °C/16 °C, relative humidity (RH) 70%). The porous foam was kept saturated with tap water for the duration of the trial. The microbiome extract described above (Section 4.2) was applied onto the spikes 1 day before inoculation of *F. graminearum* (−1 dpi). The extract of the individual treatments were sprayed onto the ears using a spray paint brush at 1–1.5 bars of pressure. Per treatment, 16 mL of extract was sprayed onto 8 ears. The ears were left to dry overnight, and 24 h later, the spikes were inoculated with *F. graminearum* conidiospores. The spores were inoculated with a spray paint brush in the same manner as the microbiome extract. Per treatment 16 mL of spore solution was applied at a concentration of $5\times 10^{5}$ spores/mL. After inoculation, the ears were kept at raised RH (95–100%) for 72 h to enhance the infection of *F. graminearum*. Multi-spectral images were taken at 14 dpi, and disease pressure was assessed using *Fv*/*Fm*.

### 4.5. Disease Assessment: Multispectral Imaging

Disease assessment was performed as described in Tan et al. (2020) [5]. A custom built multispectral phenotyping platform was used (CropReporter, Phenovation B.V., Wageningen, The Netherlands). This platform allows the visualization of diverse physiological traits in real time, based on specific absorption, reflection and emission patterns at a resolution of 6 µm. The effect of disease was assessed on the efficiency of photosystem II (*Fv*/*Fm*) (Baker, 2008). Additionally, the platform included excitation LEDs to visualize and quantify GFP tagged *F. graminearum* PH-1, enabling the assessment of fungal growth in planta. The GFP signals were corrected for autofluorescence of senescing leaves and ears, resulting in the corrected GFP value (cGFP).

### 4.6. In Planta Enrichment Cycles

**Figure 6:**
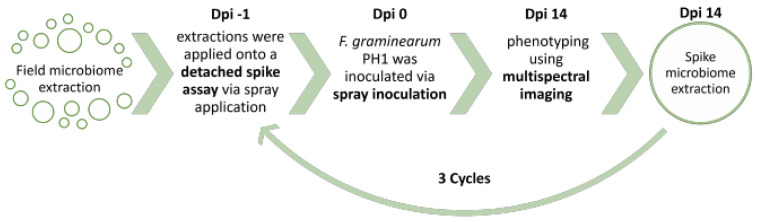
In Planta Enrichment Cycles.

Successive passaging of a plant-associated microbiome was performed in 3 identical cycles (Figure 6). The starting inoculum was the field microbiome extract applied on −1 dpi of cycle 1. At 14 dpi of cycle 1, a microbiome extraction of the cut ears was performed. Per treatment, the 4 least infected spikes were selected for extraction and a total of 20 spikelets were used. A microbiome extraction was performed as described above (Section 4.2). The extract was then re-applied onto a set of fresh cut-spikes at anthesis, initiating cycle 2. The extraction, application and inoculation were identical to the first cycle, as described in Section 4.4. At 14 dpi of cycle 2, the extraction was repeated and reapplied onto cycle 3. In every cycle, a treatment consisted of 8 spikes. In summary, the extraction/application/infection cycle was repeated 3 times, after which, a final microbiome extraction was performed and isolates were obtained.

### 4.7. Isolation of Bacteria

Subsequent to the in planta enrichment cycles, Ex-3 was selected for isolating bacteria present on the spike. With a similar methodology to the microbiome extract (Section 4.2), spikelets of Ex-3, cycle 3, were selected. The spike microbiome extract was used as the starting suspension for limiting dilution. In total, 94 isolates were selected and preserved in a glycerol stock. The spike microbiome extract was serially diluted in two different media, R2A broth (HiMedia, Mumbai, India) and a spike extract medium (SEM). SEM was prepared by boiling 50 g of spikes at anthesis in 1 L of RO water for 30 min followed by filter sterilization. The dilutions were plated in a 96-well plate by pipetting 100 µL diluted extraction per well. The plates were incubated for 14 days at 21 °C. The dilution factors were chosen in such a way that less than 50% of the wells showed visible bacterial growth after an incubation period of two weeks. The wells that showed growth in these plates were regarded as clonal cultures and subsequently handled as pure isolates. Glycerol was added to an end concentration of 25%, and isolates were stored at −80 °C.

### 4.8. Identification of Bacteria

Genotypic identification was obtained by 16S ribosomal DNA (rDNA) sequence determination and tRNA-PCR. DNA extraction was carried out by using an alkaline lysis method. This was performed by suspending a loop of plate-grown culture in an alkaline lysis buffer comprising 250 µL sodium dodecyl sulfate (10%) and 500 µL NaOH (1M), heating at 95 °C for 15 min, and carrying out a final dilution with 180 µL of sterile TE Buffer (pH = 8.0). The complete 16S rRNA sequence was determined by amplification of the 16S rRNA gene with the primers 1492r (5${}^{\prime }$-TAC GGT TAC CTT GTT ACG ACT T) and 27f (5${}^{\prime }$- AGA GTT TGA TCA TGG CTC A), and amplicon sequencing was performed via Sanger sequencing. Obtained sequences were merged and compared against the RefSeq database, using the BLAST software for species and genus assignment [71,72]. The highest identity was selected as the identified species or genus.

### 4.9. In Vitro Biocontrol Assay: Co-Inoculation

Isolates from Section 4.7 were taken out of the −80 °C and 20 µL was streaked onto a TSA plate. The plates were incubated at 25 °C for 5 days. Hereafter, a single colony was picked from the plate and inoculated into 10 mL liquid TSB medium in a 50 mL falcon. The liquid cultures were left to grow for 3 days at 28 °C at 180 rpm. The liquid cultures were centrifuged for 10 min at 3220× *g* and suspended in 1 mL PBS. The suspension was homogenized by inversion and vortexed if needed. From a fresh TSA plate, 4 circular holes were punched out using a cork borer. Holes were made in the 4 quadrants of the plate, 10 mm from the edge of the plate, and had a diameter of 8 mm. Then, 50 µL of bacterial suspension was pipetted into each of the 4 holes. Finally, an *F. graminearum* PH-1 plug originating from a 2 week old plate and 5 mm in diameter was placed in the center of every plate, with the mycelium facing down. At 3 dpi, pictures were taken from a fixed height. Pictures were analyzed with the Olympus Cell F software. Radial growth was measured from the center of the plate to the edge of the mycelium. Reduction was expressed in percentage by Mycelial Growth Reduction (*MGR*):

$$MGR=\;\left[\left(Rctr-Rtrtm\right)/Rctr\right]\;\times \;100$$

where *Rctr* = average radial growth of control plates; *Rtrt* = average radial growth of bacteria treated plates.

### 4.10. In Planta Biocontrol Assay: Detached Leaf

Leaf segments of 4 cm were cut from the tip of the leaves of 10-day-old seedlings. These leaves were placed on their abaxial surface in petri dishes containing 0.5% (*w*/*vol*) bacteriological water agar amended with 40 mg/L benzimidazole. Conidiospores were harvested from two week old *F. graminearum* PH-1 plates, and spore concentration was adjusted to $5\times 10^{5}$ spores/mL. The spore suspension (2.5 µL) was deposited in the center of the leaf segment, which was wounded using a sterile inoculation needle as previously described Ameye et al. (2015) [35]. Bacterial suspensions were prepared as described above (Section 4.9), and 2.5 µL was pipetted onto the same inoculation point. The disease progress in the detached leaves was assessed on the efficiency of photosystem II (*Fv*/*Fm*), using the multispectral phenotyping platform described above. Disease progress was assessed every 24 h for 3 days. Reduction in Necrotic Lesions on the leaf (*RNL*) was calculated as follows:

$$RNL=\;\left(Ftrt-Ffg\right)/\left(Funi-Ffg\right)\;\times \;100$$

where *Ftrt* = average *Fv*/*Fm* ratio of bacteria treated leaves; *Ffg* = average *Fv*/*Fm* ration of infected control leaves; *Funi* = average *Fv*/*Fm* ration of uninfected control leaves.

### 4.11. In Planta Biocontrol Assay: Detached Spike

The bacteria isolated in Section 4.7 were tested in a cut-spike experiment as previously described in Section 4.4. Here, the microbiome extract was substituted by a bacterial suspension. Bacteria were cultured from cryostock (−80 °C), 10 µL was plated onto TSA plates and incubated at 25 °C for 5 days. Hereafter, 1 loop (1 µL) of bacteria was inoculated in 20 mL liquid TSB medium in a 50 mL falcon. The liquid cultures were left to grow for 3 days at 28 °C at 180 rpm. On the day of the assay the liquid cultures were centrifuged for 10 min at 3220× *g* and suspended in 1 mL PBS. Just before spray application, 15 mL of PBS + tween 20 (0.05%) was added to the suspension. The suspension was homogenized by inversion and vortexed if needed.

### 4.12. Mycotoxins Analyses

The detection of deoxynivalenol (DON) and its derived metabolites and zearalenone (ZEN) was performed by a targeted LC-MS/MS using a Waters Acquity UPLC system coupled to a Quattro XEVO TQS mass spectrometer (Milford, Manchester, UK).

The mycotoxin standard of ZEN used during the LC-MS/MS analysis was purchased from Fermentek (Jerusalem, Israel). Individual mycotoxin solid standards (1 mg) of DON, 3-acetyl deoxynivalenol (3-ADON), 15-acetyl deoxynivalenol (15-ADON), deoxynivalenol-3-glucoside (DON-3G), deepoxydeoxynivalenol (DOM) and 13C-ZEN were supplied by Coring System Diagnostics (Gernsheim, Germany) as certified solutions. DON-3G solid standard was dissolved in acetonitril to a concentration of 50.2 µg/mL, all other mycotoxin solid standards were dissolved in methanol to a concentration of 1 mg/mL and were storable for a minimum of 1 year at −20 °C [73]. The standard mixture and the internal standards were prepared in methanol, stored at −20 °C and renewed monthly. Methanol (LC-MS grade) was supplied by BioSolve (Valkenswaard, The Netherlands), while n-hexane (HiPerSolv Chromanorm) and ammonium acetate were obtained from VWR International (Zaventem, Belgium). Ethyl acetate was purchased from Across Organics (Geel, Belgium), and acetic acid (glacial, 100%) was purchased from Merck (Darmstadt, Germany).

The samples were dried at 60 °C for a minimum of 48 h. The samples were then crushed, and 0.25 g of the plant tissue was spiked with 500 µg/g and 100 µg/g DOM and 13C-ZEN internal standard, respectively. One and a half milliliters of extraction solvent (ethyl acetate/formic acid (99/1, *v*/*v*)) was added to the samples. After addition, the samples were vigorously vortexed and shook on an agitator decanter overhead shaker for 1 h. The samples were then centrifuged for 15 min at 4000× *g*, and 300 µL of the supernatant was transferred to a glass tube. Subsequently, the samples were evaporated to dryness using a gentle nitrogen stream at 40 °C with the TurboVap${}^{®}$ LV (Biotage, Uppsala, Sweden). Prior to LC-MS/MS analysis, the samples were redissolved in 200 µL of injection solvent (60/40 water/methanol), vortexed for 2 min and sonicated for 30 min. Eventually, the samples were centrifuged for 10 min at 10,000× *g* in a 0.22 µm Ultrafree-MC GV centrifugal device (Merck Millipore, Burlington, MA, USA), and 150 µL of the aliquot was transferred to a new vial.

A Waters Acquity UPLC${}^{®}$ HSS T3 (2.1 × 100 mm, 1.8 µm) column was used for chromatographic separation (Waters, Manchester, UK). The temperature of the column was kept at 40 °C, and two mobile phases were applied for the analysis. Mobile phase A consisted of water/methanol/acetic acid (94/5/1, *v*/*v*/*v*), while mobile phase B consisted of water/methanol/acetic acid (2/97/1, *v*/*v*/*v*) both buffered with 5 mM ammonium acetate. The flow rate was set at 0.4 mL/min with a gradient elution program. This gradient program was as follows: 0–4.25 min, 90–70% A; 4.25–14 min, 70–27% A; 14–15.5 min, 27–1% A; 15.5–17 min, 1–90% A. The column was reconditioned for 5 min before the next injection, with a total analytical run time of 22 min. The ESI interface was used in both positive and negative electrospray ionisation mode (ESI+/ESI−). To achieve the optimal selectivity of the MS conditions, data acquisition was performed by applying selected reaction monitoring (SRM). For each target analyte, one precursor and two product ion transitions were selected. The capillary voltage was 3.3 kV and nitrogen was used as the desolvation gas. Source and desolvation temperatures were set at 130 and 200 °C, respectively. Data acquisition and processing were facilitated using MassLynx^TM^ version 4.1 and QuanLynx${}^{®}$ version 4.1 software (Waters, Manchester, UK).

### 4.13. Statistics

For plot generation, the Python [74] software version 3.8.5 and packages pandas [75], matplotlib [76] and seaborn [77] were used. For the statistical evaluation, the R software version 4.1.0 [78] and packages agricolae [79], DescTools [80] and PMCMRplus [81] were used. For multiple comparisons, normality and homoscedasticity assumptions were verified using a Shapiro–Wilk test and Bartlett’s test for homogeneity of variances. If assumptions were met, an analysis of variance (ANOVA) was conducted followed by pairwise comparisons using Dunnett’s-test for multiple comparisons with one control. A Benjamini–Hochberg correction was implemented to control the false discovery rate. If assumptions were not met a non-parametric Kruskal–Wallis test was conducted, followed by a post-hoc Dunn test. Again, a Benjamini–Hochberg correction was implemented to control the type I error when calculating multiple comparisons. For the overall comparison of the isolate performance, normality and homoscedasticity assumptions were verified using a Shapiro–Wilk test and Bartlett’s test for homogeneity of variances, and an ANOVA was conducted followed by a Fisher’s least significant difference analysis (LSD). All analyses were run at a significance level of α = 0.05.

## Figures and Tables

**Table 1:** Composition of Inoculant Carrier Mixes.

	PBS mL	Tracking Dye mL	Tween 20 (Tw) µL	CMC (S1) mg	MC (S2) mg	Metasperse^TM^ (S3) mg	Total mg
PBS	9.9	0.1	0	0	0	0	10
Tw	9.9	0.1	5	0	0	0	10.005
S1	9.9	0.1	0	1	0	0	10.001
S2	9.9	0.1	0	0	1	0	10.001
S3	9.9	0.1	0	0	0	1	10.001
Tw + S1	9.9	0.1	5	1	0	0	10.006
Tw + S2	9.9	0.1	5	0	1	0	10.006
Tw + S3	9.9	0.1	5	0	0	1	10.006

CMC: carboxylmethylcellulose (Sigma-Aldrich, St. Louis, MO, USA); MC: Methocel A15C (Sigma-Aldrich); Metasperse^TM^: Atlox Metasperse^TM^ (CRODA); tracking dye: Hydra Trak-it Red (Hydra International Ltd., Milton Keynes, UK).

**Table 2:** Identification of the isolates tested in the detached spike assay through Sanger sequencing of the 16S rRNA Gene.

Isolate	Family	Species
A5	Erwiniaceae	*Pantoea ananatis*
B3	Pseudomonadaceae	*Pseudomonas* sp.
B5	*Inconclusive*	
B8	Erwiniaceae	*Pantoea ananatis*
C11	Erwiniaceae	*Pantoea ananatis*
C3	Erwiniaceae	*Erwinia persicina*
C5	Erwiniaceae	*Erwinia persicina*
E10	Erwiniaceae	*Pantoea ananatis*
F10	Pseudomonadaceae	*Pseudomonas* sp.
H1	Erwiniaceae	*Erwinia aphidicola*
H11	Erwiniaceae	*Pantoea ananatis*
H2	Erwiniaceae	*Erwinia persicina*
H3	Erwiniaceae	*Pantoea ananatis*
H7	Erwiniaceae	*Pantoea ananatis*

## Data Availability

Not applicable.

## Supplementary Materials

The following supporting information can be downloaded at: https://www.mdpi.com/article/10.3390/toxins14030222/s1, Figure S1: Assessment of the biocontrol potential of all 94 bacterial isolates; Figure S2: Assessment of bacterial isolates to reduce mycotoxin production in a detached spike assay, full overview of mycotoxins; Figure S3: Correlation analysis comparing the different parameters, measured in the dual-culture, detached leaf and detached spike assays; Figure S4: Illustrative photographic documentation of the Dual Culture Assay; Figure S5: Illustrative photographic documentation of the Detached Leaf Assay; Figure S6: Illustrative photographic documentation of bacterial isolation via limiting dilution.
