# Supplementary material for: A Novel In Planta Enrichment Method Employing Fusarium graminearum-Infected Wheat Spikes to Select for Competitive Biocontrol Bacteria
Source: Toxins (Basel). 2022 Mar 17;14(3):222. doi: 10.3390/toxins14030222 (PMC8954829; doi:10.3390/toxins14030222)
Supplement: Supplementary file 1 [file toxins-14-00222-s001.zip › toxins-1600814-supplementary.pdf]

Supplementary Material

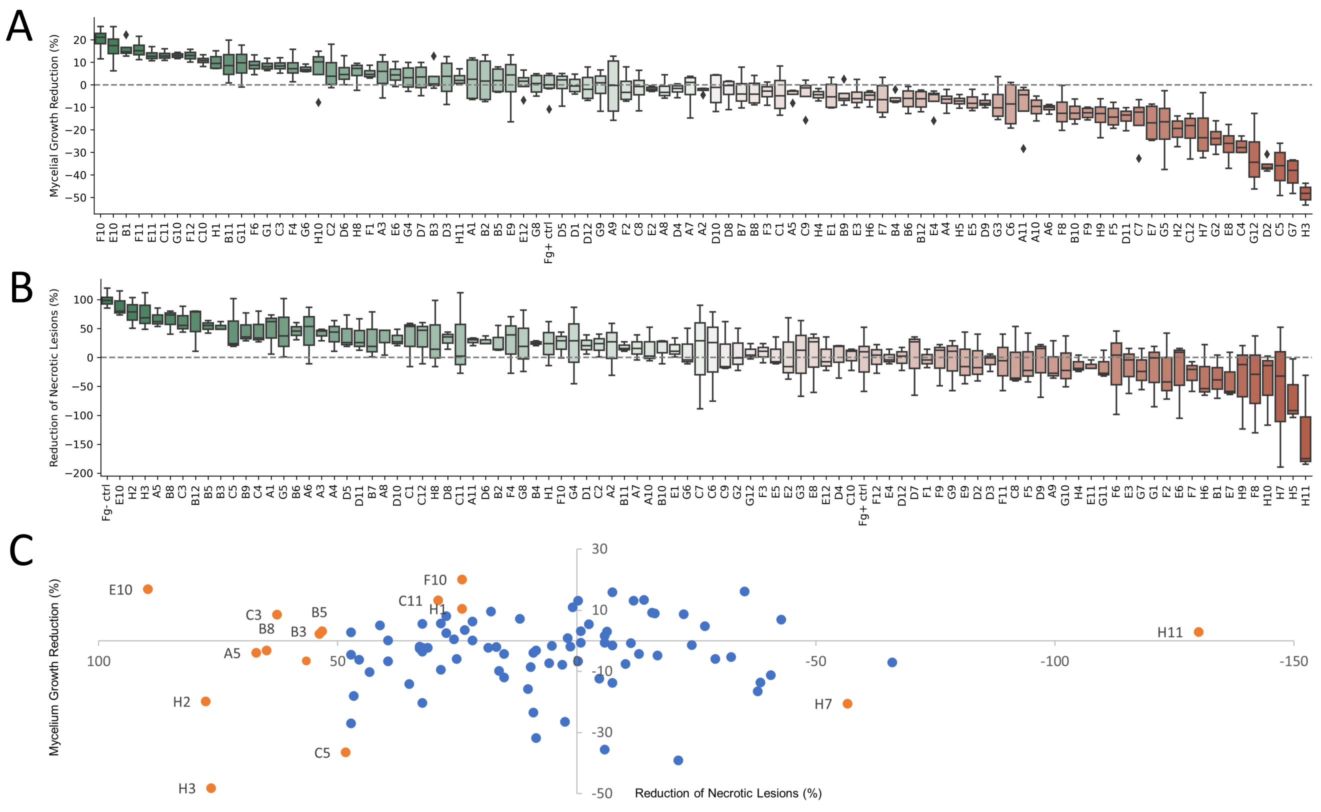

Figure S1. Assessment of the biocontrol potential of all 94 bacterial isolates. A: Reduction in mycelium growth in an in vitro dual culture assay. B: Reduction in Necrotic lesions in a detached leaf assay. C: Scatter visualising the comparison between the dual culture and the detached leaf assays

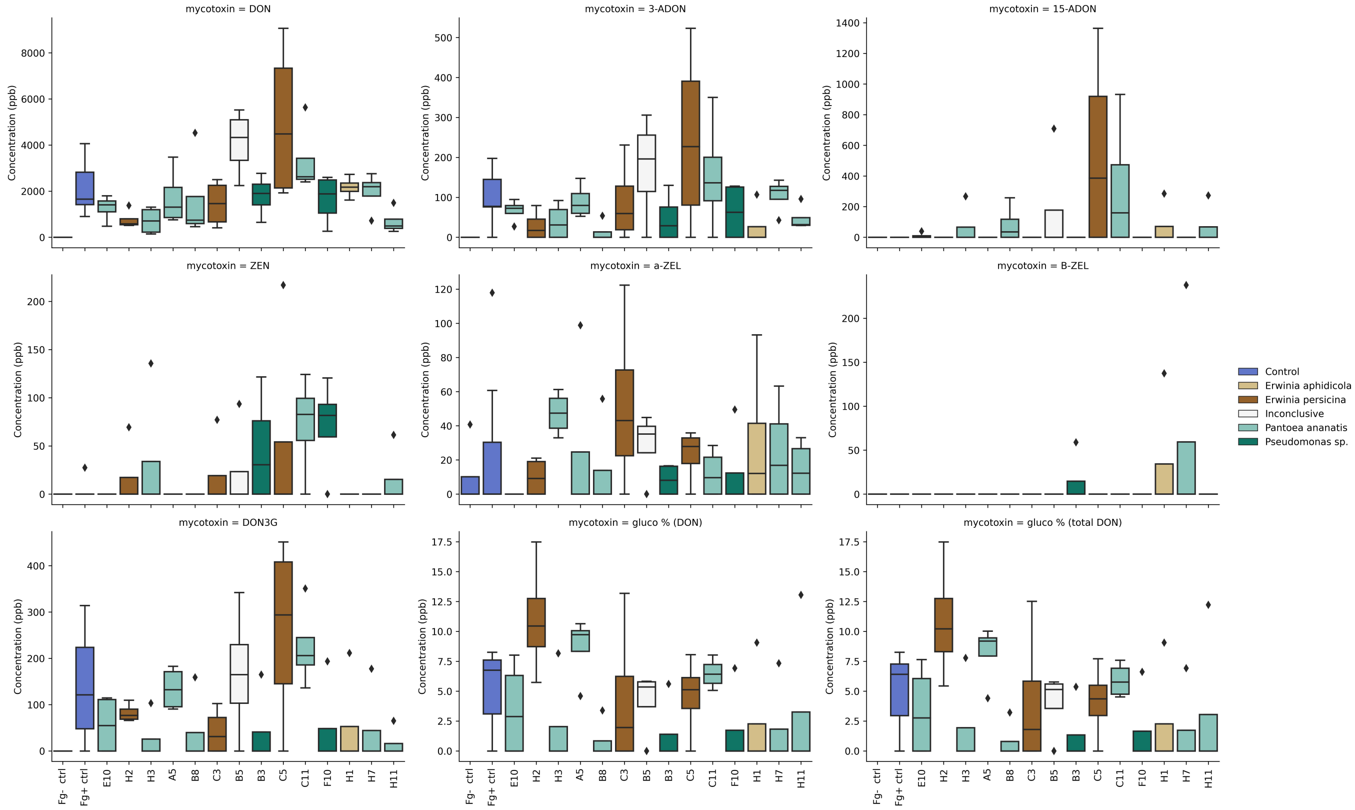

Figure S2. Assessment of bacterial isolates to reduce mycotoxin production in a detached spike assay, full overview of mycotoxins. DON: deoxynivalenol, 3-ADON: 3-acetyl deoxynivalenol, 15-ADON: 15-acetyl deoxynivalenol, ZEN: zearalenone, a-ZEL: Alpha-zearalenol, B-ZEL: Beta-zearalenol, DON3G: glucosylated DON, gluco % (DON): DON3G/ (DON + DON3G), gluco % (total DON): DON3G/ (DON + DON3G + 3-ADON + 15-ADON). All mycotoxin concentrations were measured via LC-MC/MS and concentrations are shown in µg/kg (ppb).

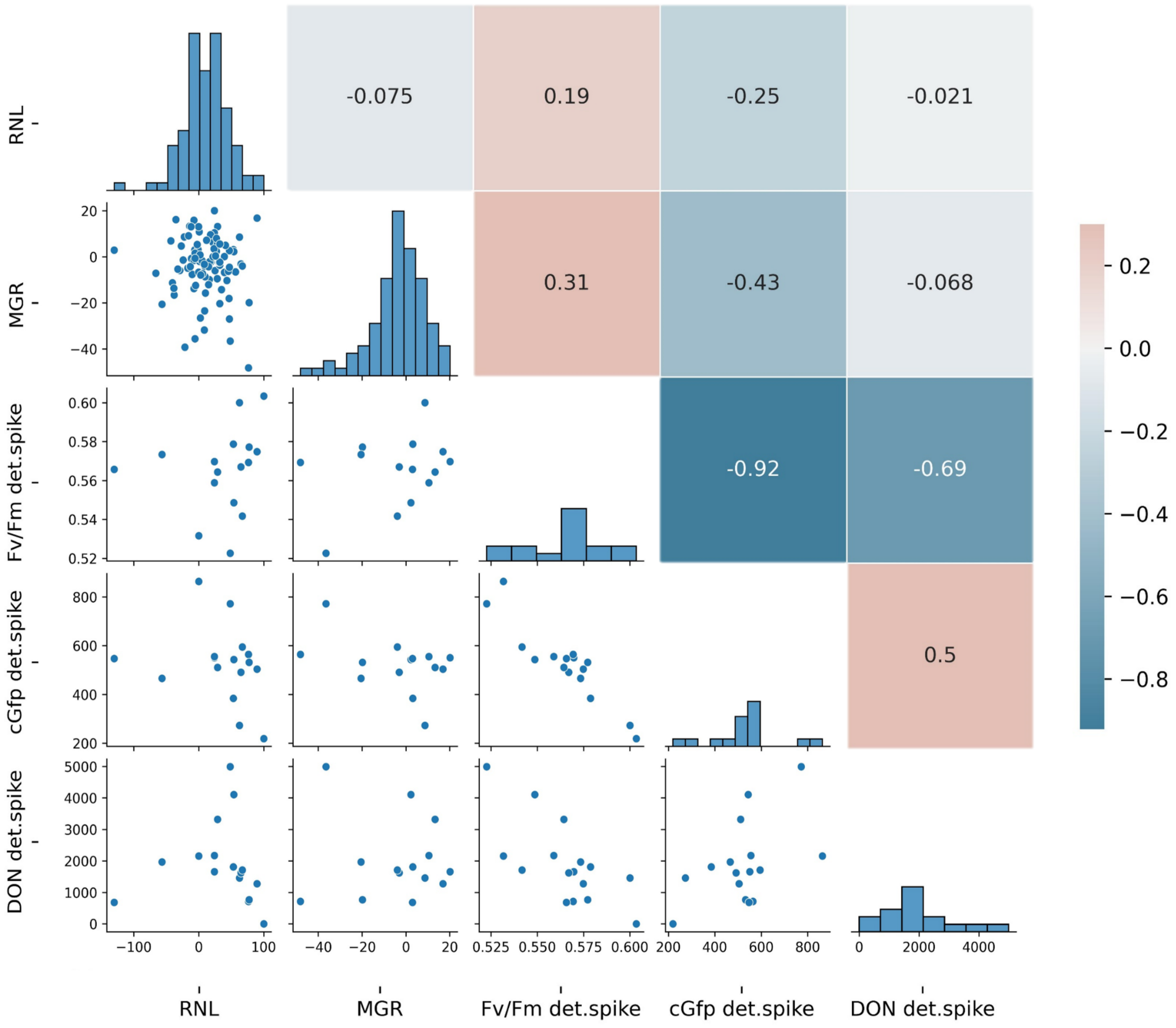

Figure S3. Correlation analysis comparing the different parameters, measured in the dual-culture, detached leaf and detached spike assays.

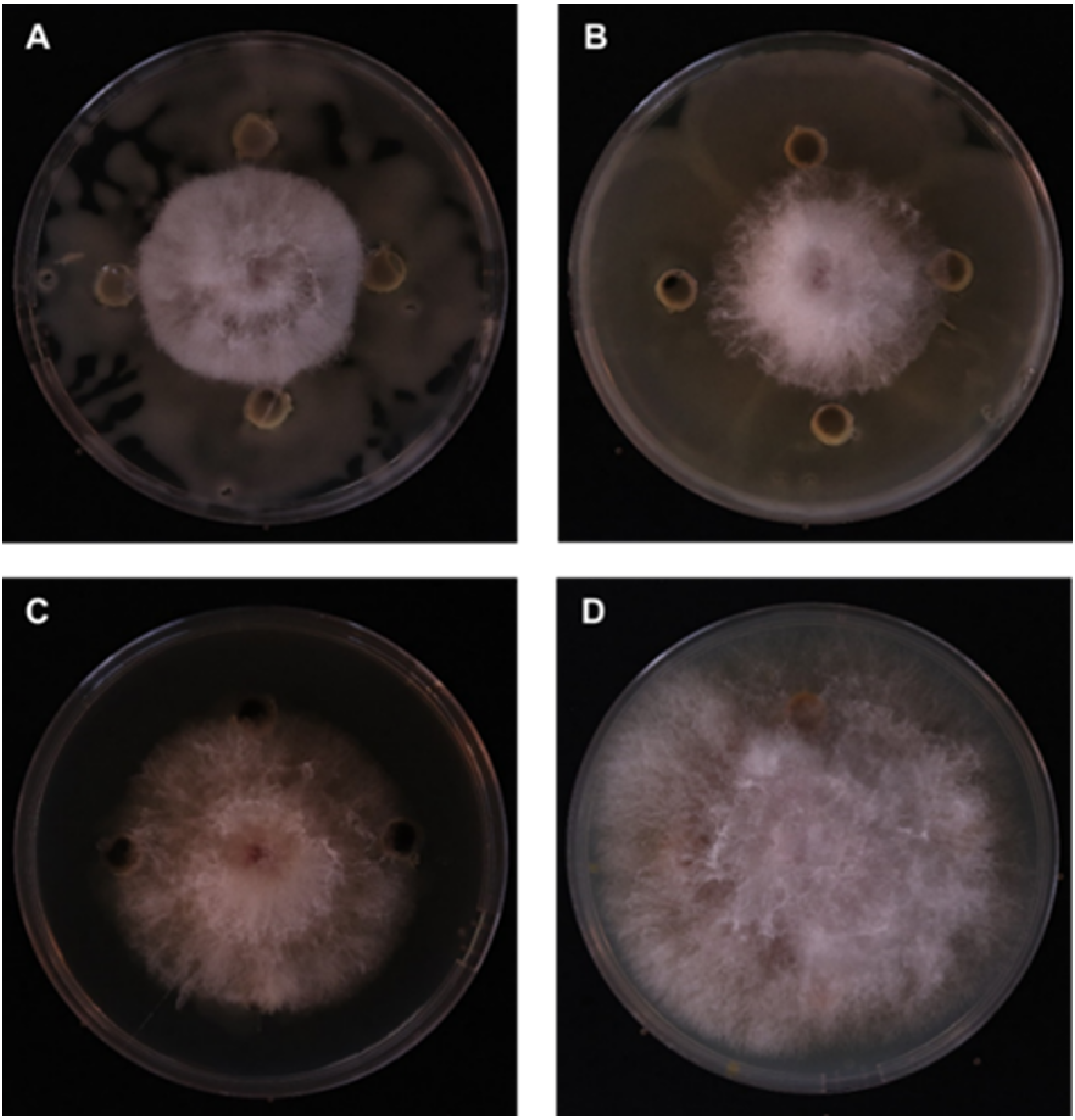

Figure S4. Illustrative photographic documentation of the Dual Culture Assay. A: *Pantoea ananatis* (E10), B: *Pseudomonas sp.* (F10), C: Fg+ ctrl, D: *Pantoea ananatis* (H3)

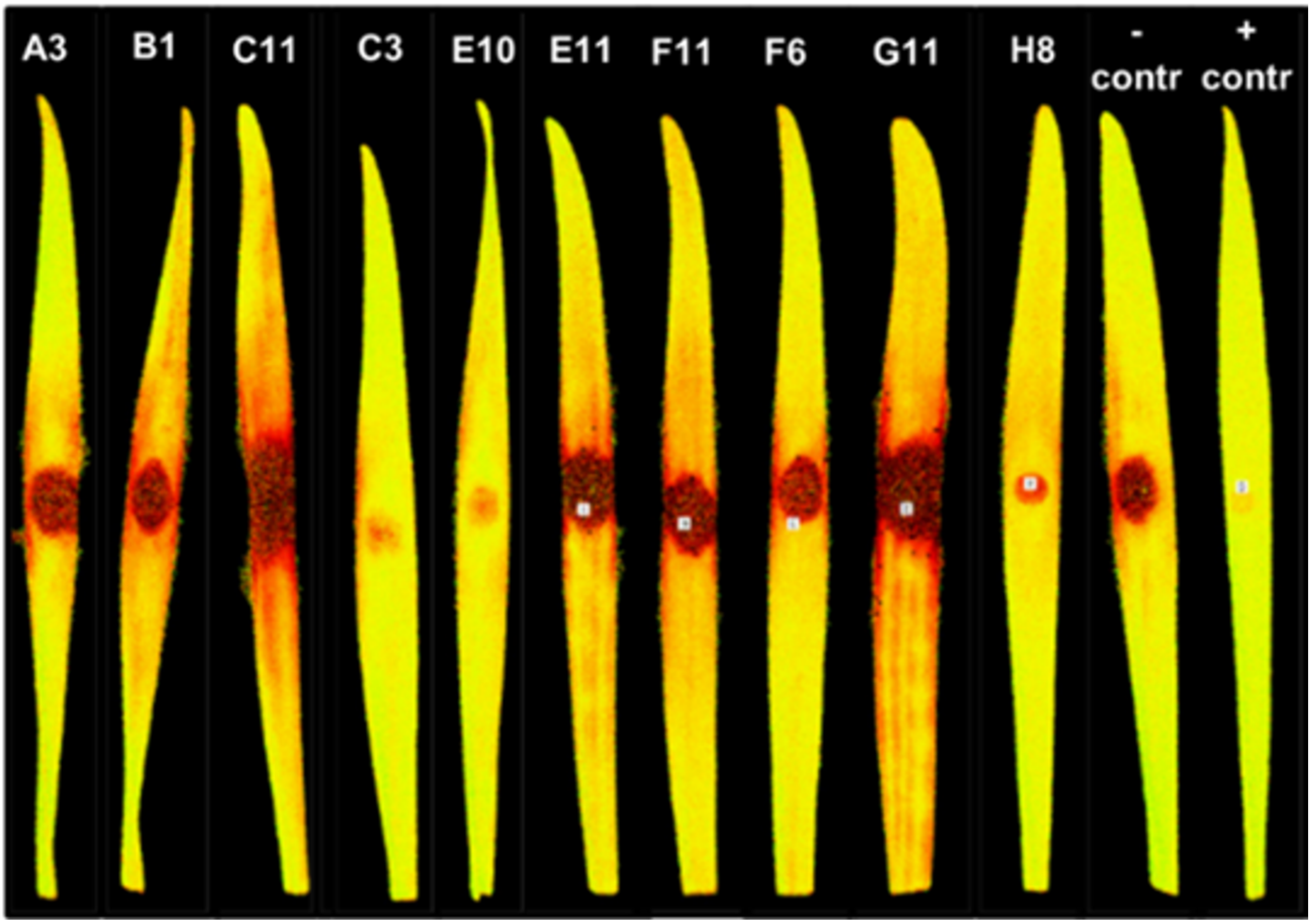

Figure S5. Illustrative photographic documentation of the Detached Leaf Assay

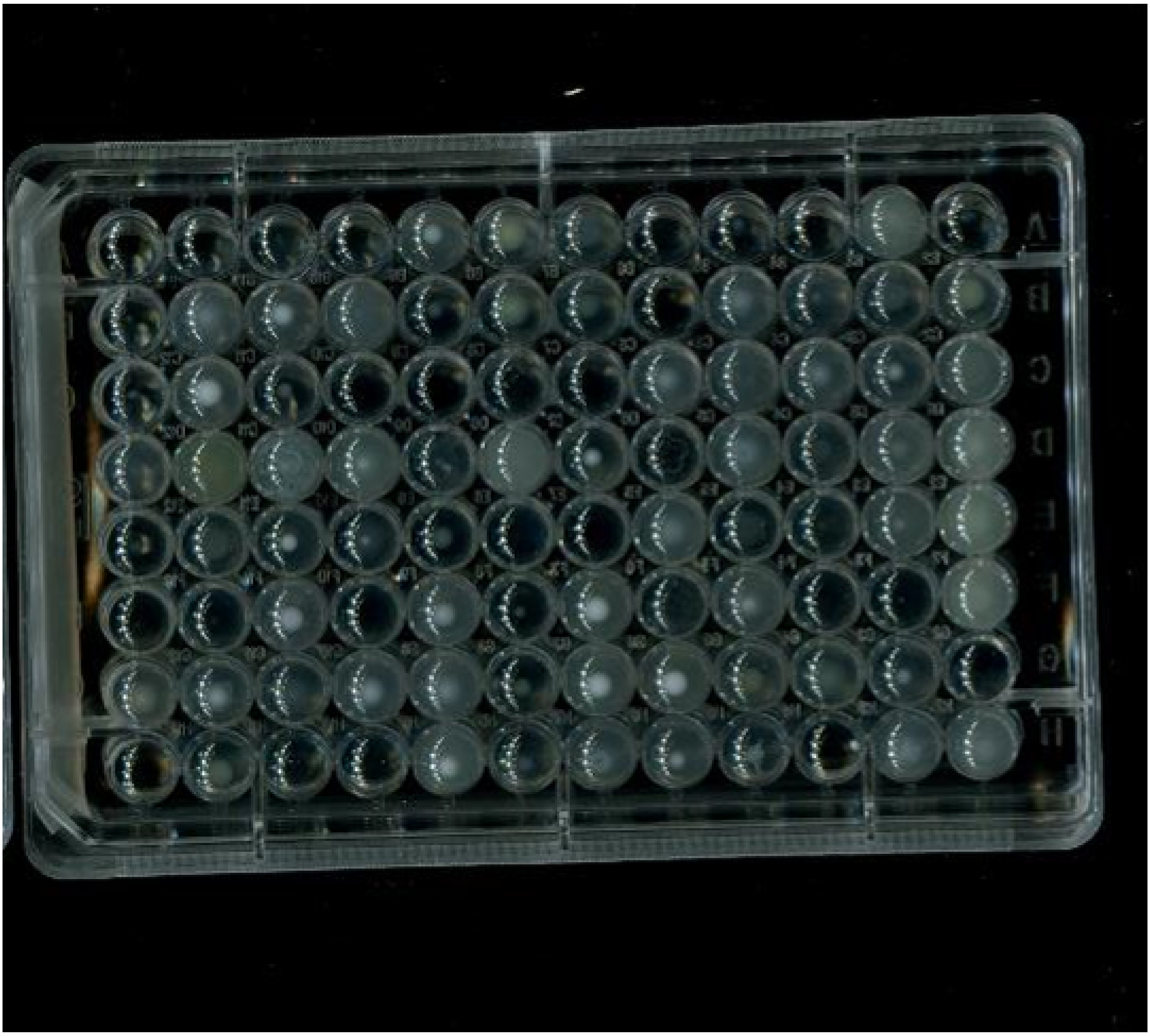

Figure S6. Illustrative photographic documentation of bacterial isolation via limiting dilution
